# Linking small‐scale fisheries co‐management to U.N. Sustainable Development Goals

**DOI:** 10.1111/cobi.13977

**Published:** 2022-10-19

**Authors:** Patrick Smallhorn‐West, Philippa J. Cohen, Michael Phillips, Stacy D. Jupiter, Hugh Govan, Robert L. Pressey

**Affiliations:** ^1^ Australian Research Council Centre of Excellence for Coral Reef Studies James Cook University Townsville Queensland Australia; ^2^ WorldFish, Jalan Batu Maung Bayan Lepas Malaysia; ^3^ Wildlife Conservation Society New York City New York USA; ^4^ Centre of Marine Socioecology, Institute of Antarctic and Marine Science University of Tasmania Hobart Tasmania Australia; ^5^ Wildlife Conservation Society, Melanesia Program Suva Fiji; ^6^ University of the South Pacific (USP), School of Law and Social Sciences (SOLASS) Suva Fiji; ^7^ Locally Managed Marine Area Network Suva Fiji; ^8^ Faculty of Science Queensland University of Technology Brisbane Queensland Australia

**Keywords:** community‐based marine management, marine protected area, coral reef conservation, Pacific, locally managed marine areas, periodically harvested closure, Por lo tanto, cuando se evalúen los impactos de la coadministración frente a los ODS, se deben considerar las metas finales, sino existe el riesgo de que haya un déficit entre las aspiraciones y el impacto, 基于社区的海洋管理, 海洋保护区, 珊瑚礁保护, 太平洋, 当地管理的海洋区域, 定期捕捞的渔场

## Abstract

Small‐scale fisheries account for 90% of global fishers and 40% of the global catch. Effectively managing small‐scale fisheries is, therefore, crucial to progressing the United Nations Sustainable Development Goals (SDGs). Co‐management and community‐based fisheries management are widely considered the most appropriate forms of governance for many small‐scale fisheries. We outlined relationships between small‐scale fisheries co‐management and attainment of the SDGs, including evidence for impacts and gaps in dominant logic. We identified 11 targets across five SDGs to which small‐scale fisheries co‐management (including community‐based fisheries management) can contribute; the theory of change by which these contributions could be achieved; and the strength of evidence for progress toward SDG targets related to various co‐management strategies. Our theory of change links the 11 SDG targets by qualifying that progress toward some targets is contingent on others being achieved first. We then reviewed 58 case studies of co‐management impacts from the Pacific Islands––a region rich in local marine governance––to evaluate evidence of where, to what degree, and with how much certainty different co‐management strategies conferred positive impacts to each SDG target. These strategies included access restrictions, permanent area closures, periodic closures, and gear and species restrictions. Although many studies provide evidence linking multiple co‐management strategies to improvements in resource status (SDG 14.4), there was limited evidence of follow‐on effects, such as improvements in catch (SDG 2.3, 2.4), livelihoods (SDG 1.2), consumption (SDG 2.1), and nutrition (SDG 2.2). Our findings suggest that leaps of logic and assumptions are prevalent in co‐management planning and evaluation. Hence, when evaluating co‐management impacts against the SDGs, consideration of ultimate goals is required, otherwise, there is a risk of shortfalls between aspirations and impact.

## Resumen

Las pequeñas pesquerías representan el 90% de los pescadores y el 40% de la pesca a nivel mundial. Por lo tanto, su manejo efectivo es crucial para el avance de los Objetivos de Desarrollo Sustentable de las Naciones Unidas (ODS). Generalmente se considera a la coadministración y la administración comunitaria de las pesquerías como las maneras más apropiadas para gestionar las pequeñas pesquerías. Perfilamos las relaciones entre la coadministración de estas pesquerías y la obtención de los ODS, incluida la evidencia de impactos y vacíos en la lógica dominante. Identificamos once metas en cinco ODS a las cuales puede contribuir la coadministración de las pequeñas pesquerías (incluyendo la administración comunitaria de las pesquerías); la teoría de cambio mediante la cual pueden lograrse estas contribuciones; y la solidez de la evidencia relacionada con varias estrategias de coadministración para el progreso hacia las metas de los ODS. Nuestra teoría de cambio conecta a las once metas de los ODS al calificar que el progreso hacia algunas metas está supeditado a que primero se logren otras metas. Después revisamos 58 estudios de caso del impacto de la coadministración en las islas del Pacífico – una región rica en gestión marina local – para evaluar la evidencia de dónde, a cuál grado y con cuánta certeza las diferentes estrategias de coadministración otorgaron impactos positivos a cada meta de los ODS. Estas estrategias incluyen restricciones de acceso, cierres permanentes del área, cierres periódicos y restricciones de equipamiento y especies. Mientras que muchos estudios proporcionaron evidencia que conecta a varias estrategias de coadministración con mejoras en el estado de los recursos (ODS 14.4), hubo pruebas limitadas de los efectos de seguimiento, como mejoras en la captura (ODS 2.3, 2.4), la subsistencia (ODS 1.2), el consumo (ODS 2.1) y la nutrición (ODS 2.2). Nuestros descubrimientos sugieren que los saltos de lógica y de suposición prevalecen en la planeación y evaluación de la coadministración.

## 【摘要】

小型渔业在全球渔民中占比90%, 而在全球渔获量中占比40%。因此, 有效管理小型渔业对实现联合国可持续发展目标 (SDG) 至关重要。共同管理和基于社区的渔业管理被广泛认为是最适合小型渔业的管理方式。本研究概述了小型渔业共同管理和实现SDG之间的关系, 包括影响的证据和主导逻辑中的空缺。我们提出小型渔业共同管理 (包括基于社区的渔业管理) 可以为5个SDG目标和11个具体目标做出贡献, 确定了实现这些贡献的变革理论, 并确定了在实现与各种共同管理策略有关的SDG目标中取得进展的证据水平。我们的变革理论通过分析目标进展是否取决于其他目标的实现, 从而将11个SDG具体目标建立了联系。接下来, 我们回顾了拥有丰富的地方海洋管理经验的太平洋岛屿的58个共同管理影响的案例研究, 以评估不同的共同管理策略对每个SDG目标产生积极影响的地点、程度和确定性的证据。这些策略包括准入限制、永久禁渔、定期禁渔以及渔具和物种限制。虽然许多研究提供了将多种共同管理策略与资源状态的改善 (SDG 14.4) 联系起来的证据, 但后续效应的证据有限, 如渔获量的增加 (SDG 2.3、2.4)、生计 (SDG 1.2) 、消费 (SDG 2.1) 和营养 (SDG 2.2) 的改善。我们的研究结果表明, 在共同管理的规划和评估中普遍存在着逻辑和假设的跳跃。因此, 在根据SDG目标评估共同管理的影响时, 需要考虑最终目标, 否则就有可能在预期和实际影响之间出现差距。**【翻译: 胡怡思; 审校: 聂永刚】**

## INTRODUCTION

The United Nations Sustainable Development Goals (SDGs) reflect a vision of inclusive progress toward human and planetary well‐being. They include goals and associated targets for ending poverty, improving food and nutrition security, and protecting natural resources, biodiversity, and ecosystems (UN, 2015). Progress toward the SDGs depends on a healthy natural resource base, which for the aquatic realm is reflected in SDG 14 “life below water,” which is to “conserve and sustainably use the oceans, seas and marine resources for sustainable development.” One of the strongest connections between human well‐being and life below water is through small‐scale fisheries, which account for 90% of all fishers and 40% of the global catch, making them the largest group of ocean users (FAO, 2022; WorldFish, 2018). Target SDG 14b specifically seeks to protect access and use rights for small‐scale fishers, who are largely seen as legitimate and effective stewards of aquatic systems, providing livelihoods and food security for approximately 492 million people, 7% of the world's population (FAO, 2022; WorldFish, 2018), and a key source of micronutrients and protein for over a billion low‐income consumers (Cohen et al., 2019).

In recent decades, there has been substantial investment in managing the types and degrees of exploitation from small‐scale fisheries, while protecting tenure rights and stewardship functions through local and collaborative governance strategies (Bender et al., 2002; Cox et al., 2010; Gurney et al., 2016). Fisheries co‐management is a governance process in which fishers, other resource stakeholders, and governments share responsibility for making and enforcing rules around resource and area use and access, in many instances with input from nongovernment organizations, civil society, and academia (Berkes, 1994; Cinner & Huchery, 2014). In practice, governance approaches range from total control by formal governments to control by local resource users (Sen & Nielsen, 1996). Co‐management is widely considered the most appropriate and effective form of governance for diverse and distributed small‐scale fisheries (Evans et al., 2011). In community‐based fisheries management (CBFM), fisheries resources are controlled by local communities (Western & Wright, 1994), yet ambient national regulations or knowledge might still be influential. In practice (e.g., in management, reports, and academic publications), the terms *co‐management*, *community‐based fisheries management*, *locally managed marine areas*, *local marine protected areas*, and *community‐managed marine areas* are frequently used interchangeably or in ways that make distinguishing them difficult. For example, *CBFM* as described in one study might encompass greater government oversight than in another study in which the term *co‐management* is used. Thus, while in specific and well‐defined governance systems, it might be possible to distinguish these terms, for the broad purposes of this study, co‐management includes any form of marine management that has some level of local control or autonomy.

Despite the increase in examples of fisheries co‐management and substantial investments by governments and nongovernmental organizations to facilitate these co‐management efforts, critical evaluation of the efficacy of fisheries co‐management to progress the SDGs remains limited. Meta‐analyses suggest that outcomes toward some socioeconomic and ecological objectives tend to be positive, although with substantial variation between cases and through time (Evans et al., 2011). These complexities, as well as the more indirect pathways toward change, present challenges for monitoring and evaluation, and we suggest programs are too often built on hopes and leaps of logic, with potentially poor understanding of causal relationships (Smallhorn‐West et al., 2020a).

Understanding how fisheries co‐management furthers the SDGs requires clarifying the causal mechanisms between management implementation and achieving various objectives. Yet, the field of impact evaluation remains nascent in its application to fisheries co‐management (Smallhorn‐West et al., 2020a). Impact is the extent to which a difference has been made, or could be made, by an intervention over and above the counterfactual condition of no intervention or a different intervention (Ferraro, 2009; Pressey et al., 2015). In some instances, it is not always ethical to apply rigorous impact evaluation techniques, such as using specific communities as controls for interventions in other communities (Pynegar et al., 2021). Yet, the fields of development and philanthropy have successfully navigated this caveat, culminating in the 2019 Nobel P being shared among three people for use of randomized control trials in alleviating global poverty (Banerjee et al., 2010). Developing even qualitative theories of change that link investments to expected impacts would be a first step toward improving many policies because they require explicit consideration of causal mechanisms and potential confounding factors (Ferraro & Hanauer, 2015; Pressey et al., 2021). This critical reflection on the links and pathways would also likely highlight poor assumptions about the impacts of co‐management. For example, it might be unrealistic to expect co‐management to improve livelihood or health outcomes unless a series of links are substantiated prior. First, the status of the resource must change, then this leads to improved yields, followed by changes in economic benefits, consumption, or both. At any point along this pathway, poor outcomes or perverse incentives could limit progress, and either inefficiencies or external factors could induce substantial delays in time or effort for each further step. Testing these assumptions and critically evaluating the material evidence for the strength of these links are, therefore, key to understanding the efficacy of fisheries co‐management.

In this study we examined relationships between small‐scale fisheries co‐management (hereafter including CBFM) and the U.N. SDGs. First, we determined SDG targets that align with established objectives of co‐management and then considered assumptions that can misrepresent progress toward these targets. We then developed a theory of change that outlines the primary links and legitimate pathways between five common co‐management strategies and specific SDG targets. Lastly, we used the South Pacific, a data‐rich region where co‐management is prevalent, as a case study to qualitatively assess the strength of evidence for these pathways between individual fisheries co‐management strategies and SDG targets.

## FISHERIES CO‐MANAGEMENT OBJECTIVES WITHIN THE SDGS

Across the 17 SDGs, 169 targets were developed that, if achieved, would mark substantial progress toward securing long‐term peace and prosperity on our planet (UN, 2015). Table 1 provides a list of 10 targets across five SDGs supported by effective and equitable fisheries co‐management. Although other targets might also be supported, these 10 targets represent those for which fisheries co‐management could drive the most progress. Of note is the overlap between target 1.4 (ensure equal rights and access to natural resources) and target 14.b (provide access for small‐scale artisanal fishers to marine resources and markets), which affects the overall count of targets and objectives. Also of note is that co‐management as a governance structure should support SDG targets 16.6 (develop effective, accountable, and transparent institutions at all levels), 16.7 (ensure responsive, inclusive, participatory, and representative decision‐making at all levels), and thereby 16.5 (substantially reduce corruption and bribery in all their forms). However, we focused on the efficacy of specific strategies within the co‐management governance framework; hence, it was impractical for us to assess the overarching structure with respect to SDG 16.

**TABLE 1:** Sustainable Development Goal (SDG) targets that effective co‐management supports in the context of small‐scale fisheries, related overarching objectives for local marine management from Jupiter et al. (2014), and analysis of common assumptions about expected management outcomes. Colours represent those commonly used for each of the five respective SGDs

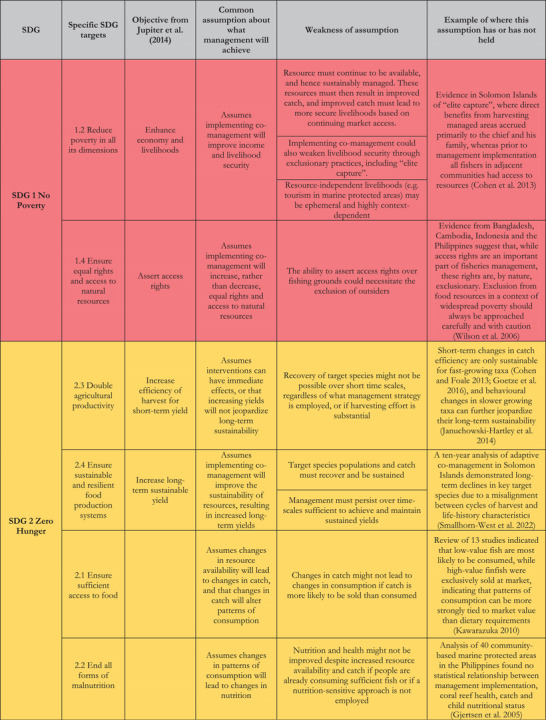
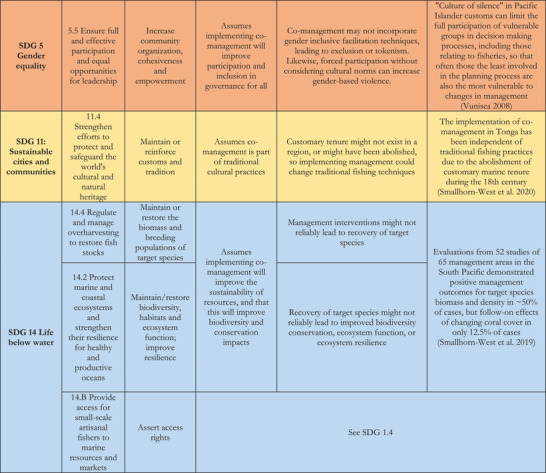

Note: reference list for tables and figures is in the Supplementary Materials.

As an initial caveat, although perhaps the most comprehensive vision of sustainable development to date, the SDG targets do not capture all elements of human well‐being. The SDG targets have been criticized as globally designed from a Western worldview and thus are thought to undermine concepts of social‐ecological resilience and human well‐being within local and regional contexts and knowledge systems (Dacks et al., 2019; Foale et al., 2011; Sterling et al., 2020). We also acknowledge our positioning within a largely Western worldview and that, although we discuss many Indigenous management systems, evidence is heavily biased toward academic outputs. We, therefore, also considered eight objectives for fisheries co‐management developed with a local marine management focus in the Pacific Island region that complement nine of the targets in Table 1 (Cohen et al., 2014; Jupiter et al., 2014).

Achieving these targets and objectives typically requires substantial changes in governance frameworks, improvements in resource status, controlling patterns of resource use, and addressing the influence and impacts of markets. Although decision makers’ conceptual models of how management drives these changes are rooted in experience and intuition, if not examined critically, they can lead to poor results stemming from poor assumptions. Table 1, therefore, alsprovides a series of assumptions that should be considered when expecting fisheries co‐management to further the SDGs, explains why these assumptions might not be valid, and gives examples of when these assumptions have not been held.

## DEVELOPING A THEORY OF CHANGE

**FIGURE 1:**
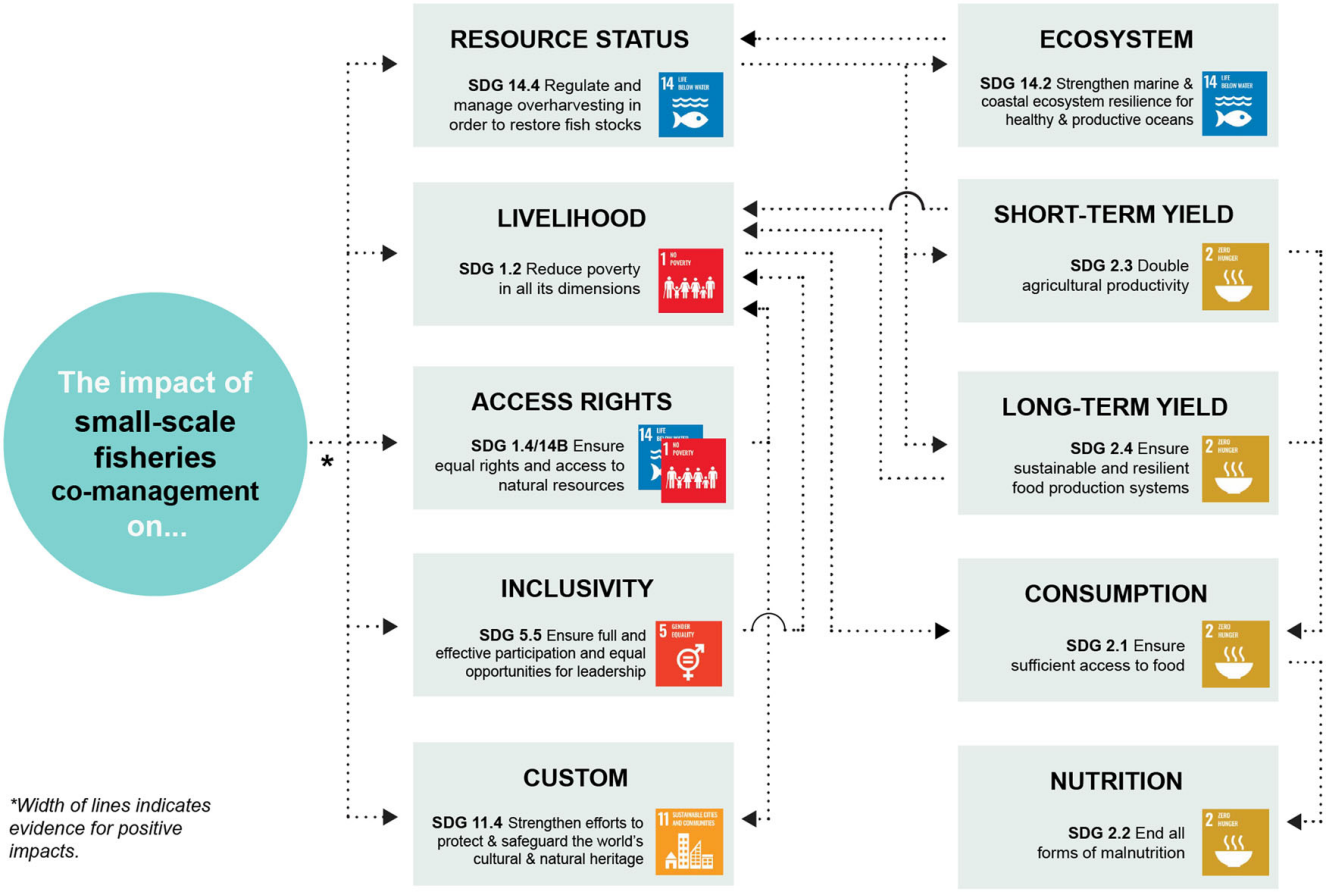
Theory of change describing the causal links (individual connections) and pathways (series of connections across multiple links) from the implementation of management strategies under fisheries co‐management to individual U.N. Sustainable Development Goal (SDG) targets (left column, direct impacts, those for which impact is not contingent on prior changes to other targets; right column, indirect impacts, those for which changes must first occur to SDG targets in the left column before impact can be achieved; colors represent those commonly used for each of the five respective SGD categories)

The path from implementing co‐management toward achieving SDG targets involves a series of causal links, direct and indirect, between actions and consequences (Ferraro & Hanauer, 2015; Pressey et al., 2021; Pressey et al., 2017). For some targets, the causal links are direct such that changes in management practices affect SDG targets without intermediate actions. For other anticipated changes, the pathways are longer, more circuitous or sequential, and contingent on changes first occurring in the other targets. For example, ensuring sufficient access to food (SDG 2.1) relies on fisheries catches being improved or sustained (SDG 2.4), which in turn relies on restoring fish stocks (SDG 14.4). Figure 1 is a theory of change that outlines proposed relationships between co‐management implementation and the 11 SDG targets in Table 1. Importantly, this theory of change does not represent a traditional impact pathway linking inputs, outputs, and outcomes to their ultimate impact. Measuring inputs (e.g., whether any management activities are in place), outputs (e.g., how many and what types of management activities there are), and outcomes (e.g., conditions within managed areas, such as changes in species abundance) can be important but do not indicate whether management makes a difference (Pressey et al., 2017). Thus, in Figure 1, each box represents an SDG target for which impacts could be achieved through co‐management. Any inputs, outputs, or outcomes are hence grouped under the box management implementation. Lines between boxes represent links (i.e., individual connections) and pathways (i.e., series of connections across multiple links) through which changes can occur. For each target, the impact is defined as changes attributable to management activities across one or a series of established indicators. As an initial caveat, this theory of change suggests only potential links and pathways (i.e., what could occur) and does not represent what actually occurs in any given circumstance. We also believe that while these links and pathways are the strongest under most circumstances, there are many others that can also occur with varying levels of impact.

We propose five SDG targets for which impacts can be achieved directly by implementing fisheries co‐management, that is, they are not contingent on prior impacts occurring in other targets (Figure 1). First, changes in the status of the resource (SDG 14.4) itself is arguably one of the fundamental goals of fisheries co‐management (Cinner et al., 2012), with follow‐on expectations for how this will affect ecosystems (SDG 14.2) and patterns of resource use (SDG 2.3 and 2.4). Livelihoods (SDG 1.2) can also be directly affected by co‐management implementation, such as when co‐management enables revenue from tourism (Brunnschweiler, 2010) or from alternative livelihood projects (Johnston et al., 2020). Access rights (SDG 1.4 and 14.b) are directly linked because the rules and regulations of co‐management decide who is, and is not, allowed to access the area and use the resources being governed (Pomeroy et al., 2007), and co‐management is frequently established to secure and bolster preexisting tenure arrangements (Foale et al., 2011; Govan, 2009). Improving patterns of inclusivity (SDG 5.5) and participation in co‐management typically occurs foremost, although not exclusively, during implementation, where the use of (or failure to use) gender‐inclusive facilitation techniques can affect SDG 5 directly and immediately (Kleiber et al., 2019). Lastly, co‐management can directly affect local and customary practices (SDG 11.4), which in some localities are reestablished, modified for contemporary purposes, or both (Johannes, 2002).

Indirect impacts on SDG targets are those contingent on changes in other SDG targets occurring as intermediate steps (Figure 1). Relationships are hence more complex and all 11 targets are involved in these pathways. Improvements in resource status (SDG 14.4) is a precursor for most other targets, for example, interacting with ecosystem state (SDG 14.2), as well as being the precondition for changes in both short‐ and long‐term sustainable yields (SDG 2.3 and 2.4). In the context of fisheries co‐management, all targets associated with SDG 2 are indirect and depend on changes in resource status or livelihoods (SDG 1.2). Patterns of catch or livelihoods (including market access) can influence patterns of consumption of aquatic foods (SDG 2.1), which in turn can affect nutrition (SDG 2.2). Livelihoods are also affected not only by how much and what is caught but also by whom and how, so that changes in access and use rights (SDG 1.4 and 14.b) and inclusivity (SDG 5.5) can affect economic situations and have further indirect affects for consumption and nutrition. Lastly, changing access and use rights can also affect customary practices (SDG 11.4), depending on how those rights are implemented and for whom (Foale et al., 2011; Jupiter, 2017). Particularly for indirect links, these represent only the most substantial pathways and there are likely many more that could and do occur.

## MANAGEMENT STRATEGIES EMPLOYED VIA CO‐MANAGEMENT

Co‐management is a form of governance under which a suite of resource management strategies can be employed in conjunction or individually (Govan, 2009; Pomeroy & Williams, 1994). These strategies are frequently locally negotiated and often reflect the reaffirmation of national regulations or a reinterpretation of customary and traditional practices. These strategies are also the principal ways in which changes in resource status, use, and access might translate to impacts for specific SDG targets. The potential impact on identified SDGs will, therefore, depend on which strategies, or combinations of strategies, are employed. Jupiter et al. (2014) outlined six resource management strategies commonly used for co‐managing small‐scale fisheries (Table 2), five of which we included here. We deliberately focused on these specific resource management strategies and did not include, but do not seek to underplay, other critical elements of co‐management, such as participation, agency, upward accountability, and other nonresource‐focused strategies. As such, alternative livelihood activities were removed as a strategy because: we considered it a component within SDG target 1.2 rather than a strategy and because many alternative livelihood strategies are not related to marine management activities.

**TABLE 2:** Management strategies within the framework of small‐scale fisheries co‐management (based on Jupiter et al. [2014])

Local management strategy	How they function	Key factors influencing their effectiveness	Caveats	Key References
Access restriction	Spatial strategy limiting who can harvest resources from within a certain area. The right or ability to restrict access is usually the first indicator of the right to manage (i.e., the ability to apply other management strategies)	Whom access is limited for, perceived legitimacy of rights, the extent to which the volume of harvest changes	Implementing access restrictions will not necessarily change the volume harvested, just who harvests it	Gelcich et al., 2012, 2017; Macintyre and Foale, 2007; Smallhorn‐West et al., 2022
Permanent closure	Spatial strategy that prohibits extractive activities within boundaries, thereby either reducing net pressure across the system or concentrating it elsewhere	Size, habitat type, distance from market access, population pressures, poaching and compliance, extent to which extraction is actually limited or instead displaced outside closures	Most locally managed closures are < 1 km^2^; permanent closures are also often configured to minimize overlap with extractive activities to avoid conflict, thereby also minimizing potential impact	Cinner et al., 2012, 2018; Edgar et al., 2014; Harrison et al., 2012; Januchowski‐Hartley et al., 2013; Russ, 2002; Russ and Alcala, 1996, 2004
Periodic closures (including temporal, nonpermanent, and rotational)	Spatial strategy whereby harvesting is allowed within an area only at certain intervals, akin to fallow agriculture on land	Same considerations as permanent closures; highly dependent on periodicity of openings, length of openings, harvest effort during openings, presence of additional restrictions during closure or opening cycles	Cycles of closure are typically shorter than required for most species to recover, and harvest efforts during openings are typically higher than what is sustainable	Carvalho et al., 2019; Cohen & Foale, 2013; Goetze et al., 2018; Januchowski‐hartley et al., 2014; Jupiter et al., 2017; McClanahan et al., 2006; Smallhorn‐West et al., 2022
Species restrictions	Size limits, bans on certain species, or bans at certain times or locations (e.g., spawning aggregations)	Species and type of restriction, as well as ability to monitor catch by fishers	Highly complex for diverse multispecies fisheries, such as those on coral reefs, so enforcement and compliance can be problematic within a local management context	Cochrane and Garcia, 2009; Foale, 1998; Hamilton et al., 2007; 2011; Léopold et al., 2013
Gear restrictions	Limits on fishing gears, typically those that are either destructive (e.g., dynamite or poison) or highly efficient (e.g., spear guns or small mesh nets)	Type and extent of gear on which restrictions are placed, ability to monitor use by fishers	Enforcement and compliance can be problematic within a local management context	Bjordal et al., 2009; Govan et al., 2008; McClanahan, 2010, 2021; McClanahan and Hicks, 2011

Note: reference list for tables and figures is in the Supplementary Materials.

## EVIDENCE OF CO‐MANAGEMENT IMPACTS ON FURTHERING U.N. SDGS IN THE SOUTH PACIFIC

We built on a literature review by Smallhorn‐West et al. (2020a) to qualitatively assess the strength of the evidence linking individual co‐management strategies to positive SDG impacts from the South Pacific, including the following countries and territories: Cook Islands, Fiji, French Polynesia, New Caledonia, Niue, Samoa, American Samoa, Tonga, Tuvalu, Vanuatu, and Wallis and Futuna. Full details of the literature search methodology are available in Smallhorn‐West et al. (2020a). Briefly, 58 articles were examined that quantified the socioeconomic or ecological impacts of co‐management in the region. For each article, the number, type (e.g., change in resource status, change in income from catch), and direction (i.e., positive or negative) of impacts were recorded, as well as the management strategy employed. We expanded the search to include more recent articles; Papua New Guinea and the Solomon Islands; and species and gear restrictions. We then qualitatively assessed the strength of evidence for a positive effect of each co‐management strategy on each SDG target, subject to several caveats. First, ideally, three things would be assessed: strength of evidence, the direction of effect, and effect size; however––we evaluated only the strength of evidence for a positive effect based on the number of studies reporting positive outcomes. This was because of difficulties quantifying what counts as sufficient evidence across multiple SDG targets and co‐management strategies; potential biases in the literature toward positive results; and the broad range of indicators preventing quantitative assessments or meta‐analysis on effect size. Our evaluation is, therefore, based on our interpretations of how the published literature fits within the presented theory of change, and some papers individually provided more weight than others did combined.

**FIGURE 2:**
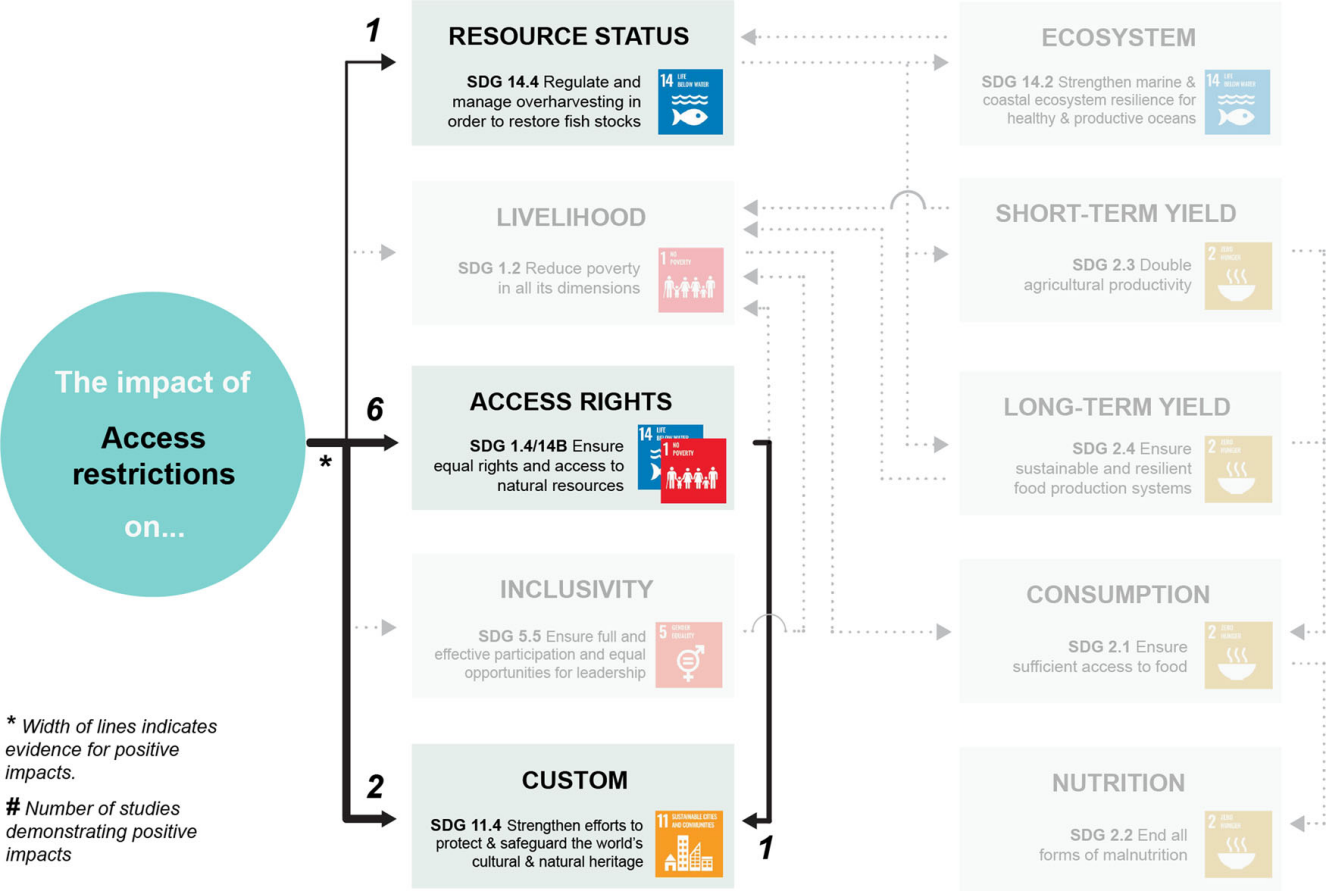
Theory of change describing the strength of evidence for positive impacts from fishing access restrictions toward achieving 11 U.N. Sustainable Development Goal targets (gray, targets for which no evidence of positive impacts could be found). The width of each link (individual connection) indicates the strength of evidence for a positive impact based on the authors’ interpretation of the literature. The numbers indicate the number of studies demonstrating positive impacts. Note: reference list for tables and figures is in the Supplementary Materials.

**FIGURE 3:**
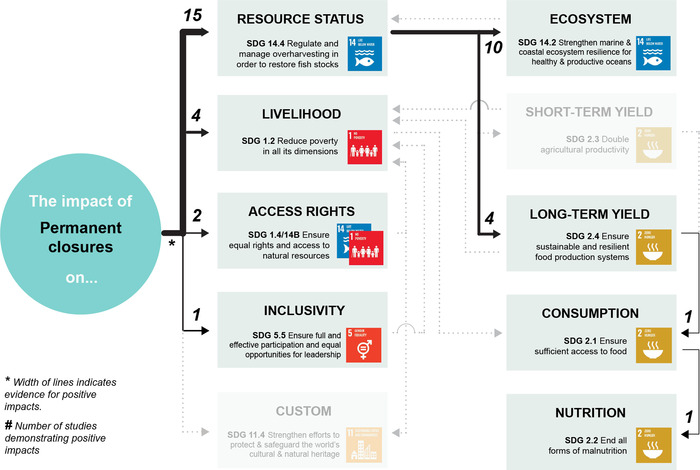
Theory of change describing the strength of evidence for positive impacts from permanent closures toward achieving 11 U.N. Sustainable Development Goal targets (gray, targets for which no evidence of positive impacts could be found). The width of each link (individual connection) indicates the strength of evidence for a positive impact based on the authors’ interpretation of the literature. The numbers indicate the number of studies demonstrating positive impacts Note: reference list for tables and figures is in the Supplementary Materials.

**FIGURE 4:**
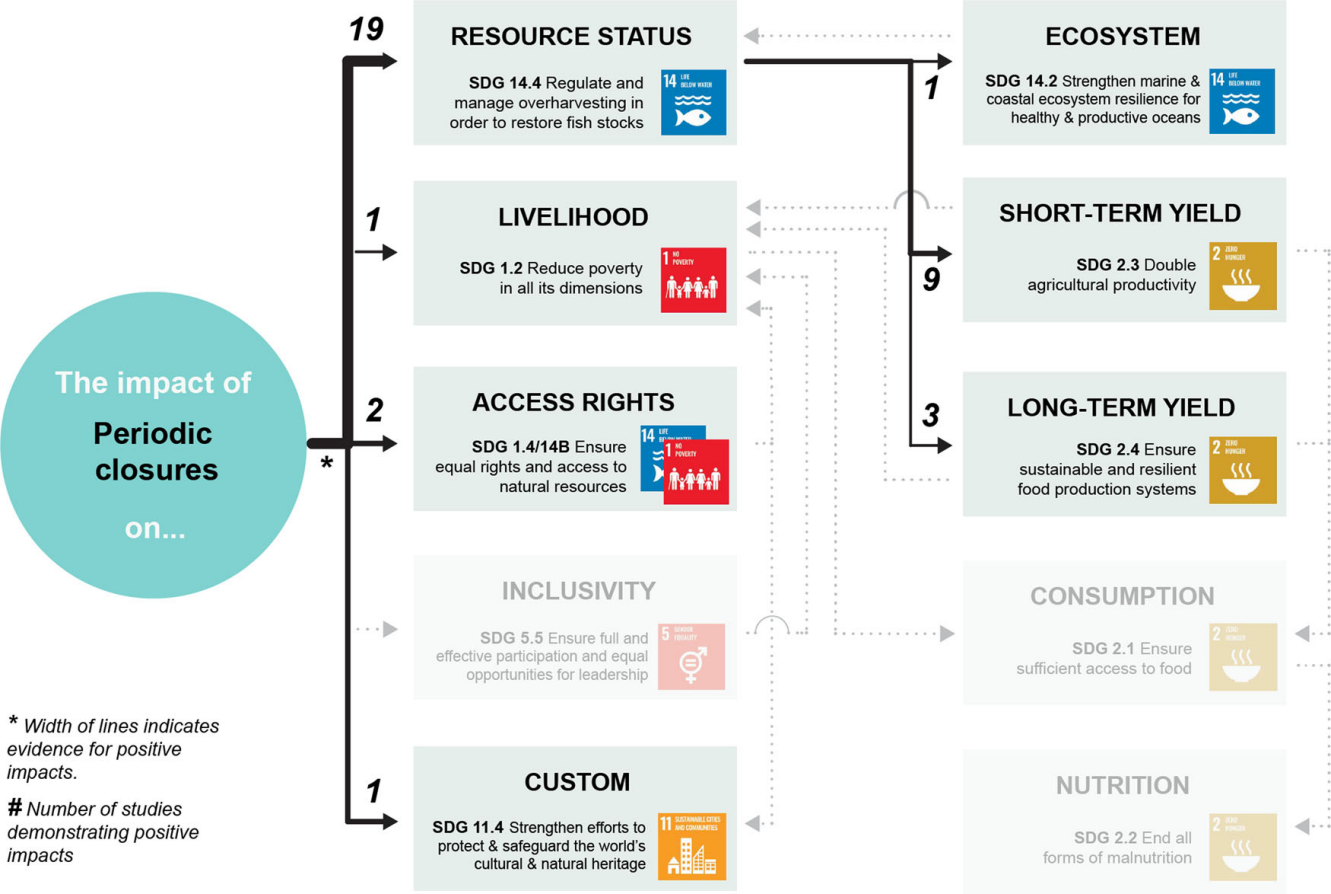
Theory of change describing the strength of evidence for positive impacts from periodic closures (including temporal, non‐permanent, and rotational closures) towards achieving 11 U.N. Sustainable Development Goal targets (gray, targets for which no evidence of positive impacts could be found). The width of each link (individual connection) indicates the strength of evidence for a positive impact based on the authors’ interpretation of the literature. The numbers indicate the number of studies demonstrating positive impacts. Note: reference list for tables and figures is in the Supplementary Materials.

**FIGURE 5:**
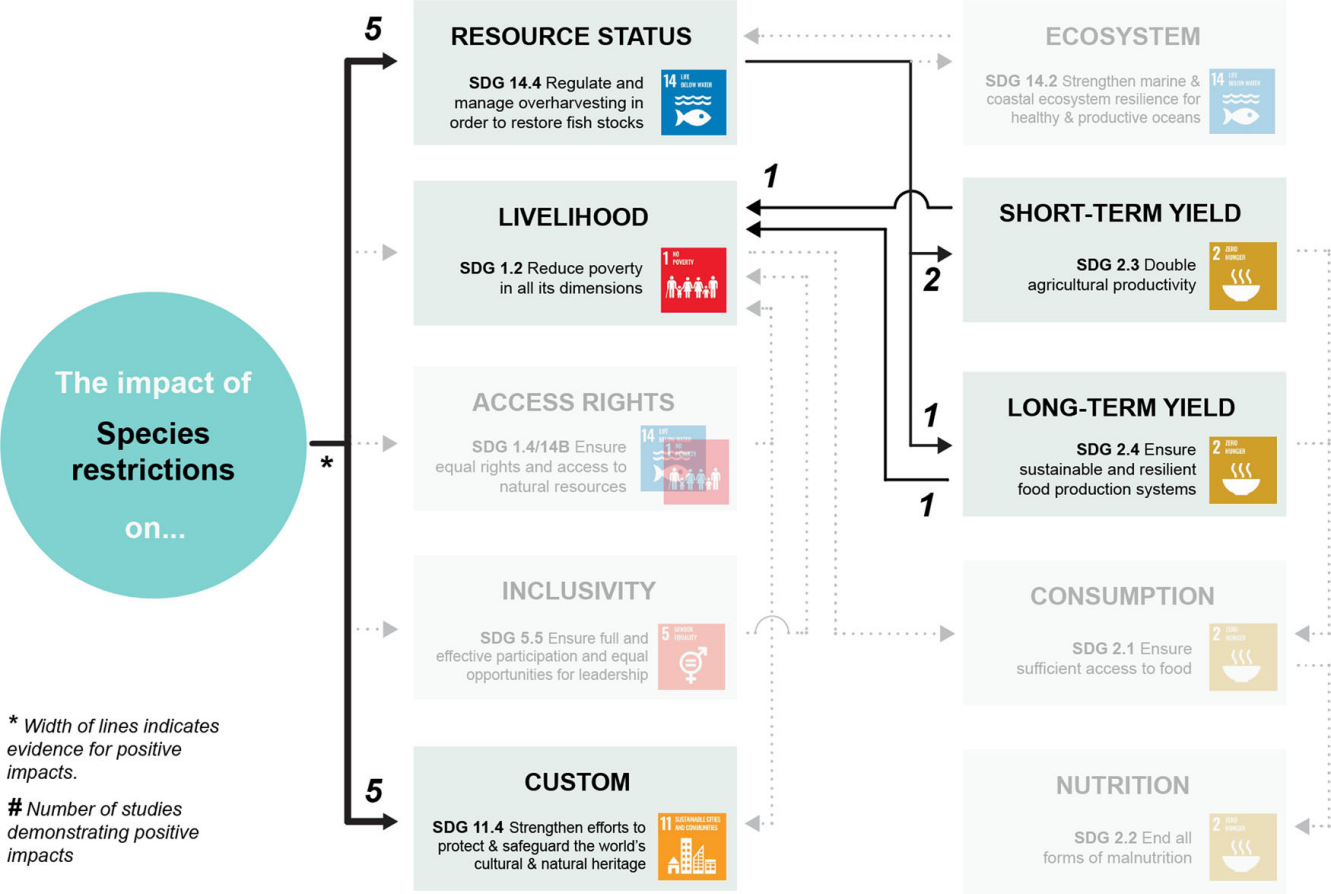
Theory of change describing the strength of evidence for positive impacts from species restrictions towards achieving 11 U.N. Sustainable Development Goal targets (gray, targets for which no evidence of positive impacts could be found). The width of each link (individual connection) indicates the strength of evidence for a positive impact based on the authors’ interpretation of the literature. The numbers indicate the number of studies demonstrating positive impacts. Note: reference list for tables and figures is in the Supplementary Materials.

**FIGURE 6:**
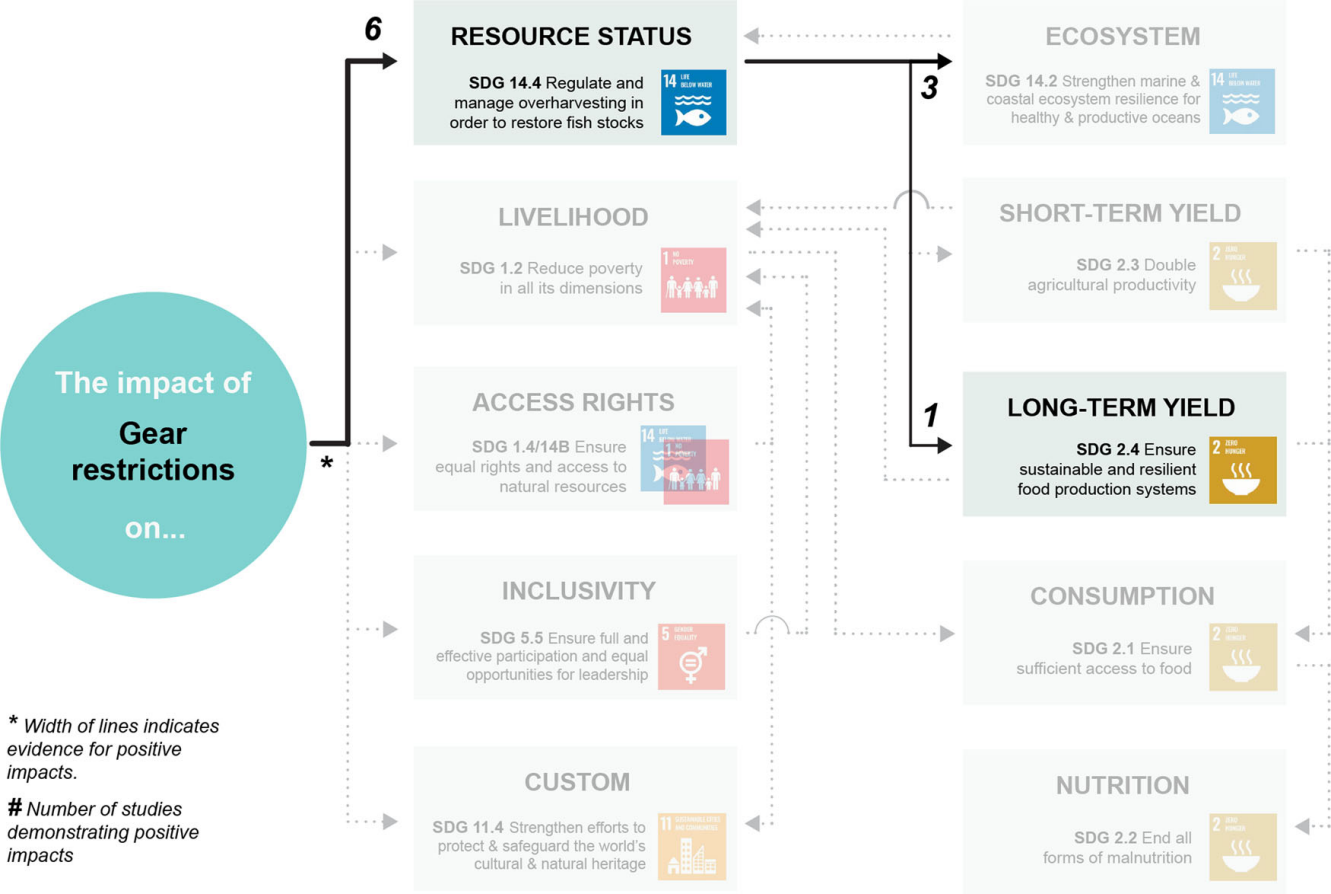
Theory of change describing the strength of evidence for positive impacts from gear restrictions towards achieving 11 U.N. Sustainable Development Goal targets (gray, targets for which no evidence of positive impacts could be found). The width of each link (individual connection) indicates the strength of evidence for a positive impact based on the authors’ interpretation of the literature. The numbers indicate the number of studies demonstrating positive impacts. Note: reference list for tables and figures is in the Supplementary Materials.

Appendix S1 maps evidence gaps by summarizing the number of studies included for each combination of co‐management strategy and SDG target. Figures 2, 3, 4, 5, 6 then represent theories of change for each co‐management strategy listed above, with relevant literature included within each SDG target. The width of each connection represents the strength of evidence for a positive link between co‐management implementation and SDG targets for direct impacts and between SDG targets for indirect impacts. Overall, the clearest findings were a major focus on changes in resource status, and most studies focused on direct impacts of co‐management; far fewer examined pathways to indirect impacts. We found only one study in the region that links patterns of consumption and nutrition to co‐management that fitted our selection criteria (Aswani & Furusawa, 2007). Many studies do link various SDG targets independently of their relation to co‐management, such as patterns of consumption and noncommunicable diseases (Anderson, 2013; Kronen et al., 2004; Lyons et al., 2020). However, we could not investigate all links and pathways between these SDG targets that were not directly associated with co‐management.

### Access restrictions

Access restrictions (Figure 2) have long been practiced in the South Pacific region as a way of recognizing local tenure arrangements and supporting local governance (Johannes, 1978, 2002). The right or ability to restrict access and use is usually the first indicator of the right to manage a particular resource and, hence, the right to apply the other strategies listed below. As such, there is strong evidence linking access restrictions to SDG 1.4, 14.b, and 11.4. Johannes (1978, 2002) argued that these restrictions, as well as other traditional practices, such as periodic closures, evolved as a social response to marine resource scarcity. However, Foale et al. (2011) suggested that, due to low population pressures, these restrictions instead evolved to manage relationships between social groups rather than sustain food security through fisheries. Because changing resource status relies on changing patterns of resource use, as well as access restrictions, these restrictions influence only who harvests and not necessarily the volume harvested (Polunin, 1984). Overall, there is little evidence of their effectiveness at driving change toward SDG 14.4 or the flow‐on SDG targets. There was one instance of evidence of improvements in target fish productivity due to implementing restricted access zones in conjunction with adjacent permanent closures (Smallhorn‐West et al., 2022).

### Permanent closures

Permanent closures (Figure 3), including no‐take reserves that may be situated within marine protected areas (MPAs) and locally managed marine areas, are employed for fisheries management and marine conservation worldwide (Edgar et al., 2014; Pressey et al., 2015; Waldron et al., 2020) and are thus the most visible, although not the most common, co‐management strategy in the South Pacific region. Most studies quantifying fisheries co‐management impacts in the South Pacific have correspondingly assessed changes associated with permanent closures. There is much evidence that these approaches do drive substantial improvements in resource status within boundaries (e.g., Bartlett et al., 2009; Bonaldo et al., 2017; Smallhorn‐West et al., 2020b) and can result in spillover of larvae and adults into adjacent areas (e.g., Almany et al., 2013; Harrison et al., 2020; Januchowski‐Hartley et al., 2013). These changes can also have flow‐on benefits for ecosystem status (Bonaldo et al., 2017; Bonaldo & Hay, 2014; Dell et al., 2015), and there is some evidence for changes in long‐term patterns of catch (Clements et al., 2012; Pascal, 2011). However, permanent closures do not necessarily reduce net pressure across a fishing ground, but instead can displace it from one area to another (Vaughan, 2017), so there is no evidence of short‐term improvements in yield. There is some evidence for improving livelihoods from permanent closures. For example, Pascal and Seidle (2013) examined the economic effects of MPAs in 10 villages in Fiji and Vanuatu and found positive cost–benefits ratios across five ecosystem services (subsistence fisheries, commercial fisheries, tourism, bequest value, and coastal erosion). Evidence for improving access and custom targets is weak because permanent closures typically restrict access and are generally not part of customary management practices in the region (Foale et al., 2011; Love, 2021). A notable exception is in Tonga, where local tenure arrangements were abolished in the 19th century and permanent closures are being expanded as part of efforts to reestablish community‐based management through the development of Special Management Areas (Smallhorn‐West et al., 2020c). Only one study (Cakacaka et al., 2010) examined patterns of women's and men's participation in management following the establishment of permanent closures. Although results were positive, participation was quantified based on attendance and not on whether women and men had equal opportunities to understand, share information, provide input, or be understood (Kleiber et al., 2019). Lastly, one study compared indicators of nutritional intake (e.g., grams of protein and fat) and human health (e.g., anthropometric measurements) between villages with and without no‐take closures (Aswani & Furusawa, 2007). However, while this study represents an important step in quantifying these patterns, the results were purely correlative and hence a link could not be established to changes in patterns of catch or resource status.

### Periodic closures

Periodic closures (including temporal, nonpermanent, and rotational closures) (Figure 4) are the main component of many co‐management systems in the South Pacific region (Foale et al., 2011; Johannes, 1978, 2002). They are highly variable, falling on a spectrum from predominantly closed to those regularly harvested (Cohen & Foale, 2013; Govan, 2009; Jupiter et al., 2014). Their impact is hence highly dependent on cycles of opening and closing. Much research highlights short‐term improvements in resource status (e.g., Carvalho et al., 2019; Cinner, 2005; Goetze et al., 2018), as well as in short‐term yields driven by changes in fish naiveté when they are left alone for periods of time (Cohen et al., 2013; Cohen & Alexander, 2013; Januchowski‐hartley et al., 2014). However, increases in target species abundance are typically observed only prior to harvesting (Smallhorn‐West et al., 2020a). There are also long‐term risks associated with misaligned cycles of harvesting and the life histories of target species, resulting in steady declines of target species over multiple cycles (Smallhorn‐West et al., 2022). Lastly, because of their traditional origins, these approaches provide substantial advances toward securing access rights and maintaining customs for many small‐scale fishing communities (Foale et al., 2011).

### Species restrictions

Impacts of restrictions on species (Figure 5) harvested, or gears used for harvesting, are highly contingent on both the type of species or gear and what the restriction entails. Improving SDG targets in fisheries that utilize many species and gear types, such as in the South Pacific region, further increases the diversity of potential outcomes (McClanahan et al., 2015). Nevertheless, there is good evidence that species restrictions are able to improve the resource status of harvested species (Figure 5). For example, Almany et al. (2013) and Hamilton et al. (2011) examined outcomes of banning the catch of species during spawning, resulting in a 10‐fold increase in target species density (Hamilton et al., 2011) and substantial larval contributions to following generations (Almany et al., 2013). There is also strong evidence that formally implementing species bans supports community cultural practices, such as totemism, if these practices are already occurring within the community (Veitayaki, 1995). Lastly, one study (Léopold et al., 2013) demonstrated positive links between sea cucumber (*Holothuria scabra*) abundance, catch, and annual returns in New Caledonia following harvest restrictions, the longest causal pathway for which evidence could be found in the region. We acknowledge current work in Fiji (and other Pacific Island countries and territories) to update and incorporate species‐specific size limits into co‐management strategies (e.g., Prince et al., 2021), although these have not yet been quantitatively tested relative to specific outcomes.

### Gear restrictions

We identified seven studies from the South Pacific region examining the impacts of gear restrictions, the fewest for any management strategy (Figure 6). Most studies also used different framing than for spatial restrictions, focusing on patterns of catch for various gears and damages caused by various gears, rather than specific assessments of outcomes associated with each gear type. Nevertheless, these studies suggest that gear restrictions associated with co‐management can be effective for managing the status of target species. Three of these studies (Cinner & McClanahan, 2006; McClanahan et al., 2006; 2008) examined the same traditionally managed areas in Papua New Guinea that combine gear restrictions with periodically harvested closures and showed increased biomass and an average size of target species. Veitayaki (1995) reported on destructive fishing gears in the Pacific Island region, with examples of negative consequences for coral reef ecosystems. McClanahan et al. (2008) compared patterns of yield and catch per unit effort among fishing nets, fishing lines, and spearguns and found strengths and weaknesses for all three types in terms of use and conservation of resources.

## DISCUSSION

Co‐management is often deemed an appropriate governance system for small‐scale fisheries given their dispersed, diverse, and dynamic nature, as well as its ability to adjust to local circumstances and adapt through time. These characteristics of small‐scale fisheries are often considered ungovernable through other, more centralized, governance models (Jentoft, 1989; Jentoft & Chuenpagdee, 2009; Khan & Neis, 2010). Nonetheless, while the expectations for co‐management to deliver a whole suite of outcomes are high, we found that this is not necessarily met with sufficient levels of evidence. We suggest that, based on current evidence from the South Pacific region, co‐management is primarily useful for securing fisher access rights and improving the stock status of particular marine resources (e.g., biomass, resilience, and/or productivity). Because many SDGs are contingent on a sustainable resource base, progress toward SDG 14 should be expected to drive progress toward other indirect SDG targets. Yet, progress toward many of the SDGs will also require simultaneous investments in improved services, food and nutrition security, rural development, reduced corruption, and government support, in addition to investments into co‐management. There is currently only limited evidence linking small‐scale fisheries co‐management to improved livelihoods, consumption, or human health in the South Pacific region. This identified gap is likely due to the increasing difficulty of quantifying indirect impacts further along the theory of change because these SDG targets are also influenced by a host of other factors that make measuring the contribution of co‐management especially challenging. Likewise, factors affecting resource governance outside the remit of co‐management, such as market forces, institutional capacity, or accountability of governance institutions, may have impacts that overshadow the influence of co‐management (Coastal Fisheries Working Group, 2019).

Future research on the impacts of small‐scale fisheries co‐management should emphasize filling knowledge gaps for indirect SDG targets, rather than continuing to prioritize measuring changes in resource status (e.g., SDG 14.4). We acknowledge that formal impact evaluations can be prohibitive in cost and expertise and must, therefore, be balanced with the realities of resource availability. Nevertheless, given the growing recognition of small‐scale fisheries contribution to food and nutrition security (Cohen et al., 2019; Hicks et al., 2019; Kawarazuka, 2010), strengthening understanding of how co‐management supports these targets remains critical. Quantitative evaluations are, therefore, needed to inform policy; otherwise, alternate policies and practices that might have more impact may not be adopted.

Several of the identified evidence gaps can be filled by more qualitative research that did not meet the quantitative inclusion criteria in our case study, particularly for SDG 5.5 (inclusivity) and SDG 11.4 (customs). For example, despite women's contribution to small‐scale fisheries often being invisible, ignored, or unrecognized, there is a substantial and growing literature documenting their crucial role in the sector (Lawless et al., 2021, 2022; Mangubhai et al., 2022; Mangubhai & Lawless, 2021; Thomas et al., 2021). This contribution extends well beyond subsistence narratives from specific fisheries (i.e., gleaning) and includes activities across a wide range of habitats and sections of the value chain (Grantham et al., 2020). The co‐management of small‐scale fisheries, therefore, has great potential to help, or hinder, progress toward SDG 5, depending on the processes through which it is implemented. For example, co‐management can exacerbate existing inequalities when local power structures are highly asymmetrical through the process of elite capture (Warren & Visser, 2016), resulting in potential regression away from SDG 5. Yet, approaches also exist to guard against this, such as using gender‐inclusive facilitation techniques (Kleiber et al., 2019). This growing body of literature on gender principles within small‐scale fisheries co‐management, much of which originates from the Pacific region, suggests that the process of co‐management, rather than any one individual strategy, at the very least, has great potential to reduce gender inequities, even if the extent to which this is occurring across the region in practice remains unclear.

The quantitative criteria used in our case study also limited the inclusion of many lessons reported by Pacific authors on the importance of Indigenous knowledge for managing small‐scale fisheries (Benyei et al., 2020; Kakuma & Kitolelei, 2018; Vave, 2022; Veitayaki, 2000, 2002; Veitayaki et al., 2004) and hence furthering SDG 11.4 (strengthening efforts to protect and safeguard the world's cultural heritage). For example, Veitayaki (2002) presents the knowledge, wisdom, and experiences of Indigenous Fijian communities that relate to sustainable resource management, including fisheries. Although the structure of information by Veitayaki (2002) made it difficult to include in our analyses, it nevertheless provides evidence of how local marine management in the region is tightly bound to local customs; hence, supporting one should support the other. Likewise, Vave (2022) outlined how the central tenet of community‐based natural resource management in the Pacific is that it is part of Indigenous culture and tradition. In many circumstances, formal co‐management arrangements are developed from existing local and customary tenure, thereby supporting SDG 11.4. Yet, in some circumstances, Indigenous peoples and local communities have been required to give up certain rights (e.g., the ability to enforce tenure) in order to secure formal legal recognition of co‐management arrangements (Mangubhai et al., 2020). In these instances, adopting formal co‐management arrangements could potentially weaken, rather than strengthen, cultural heritage.

Society can often ask too much from co‐management, expecting it to solve too many problems. One appeal of co‐management is that it can be used alongside other governance tools to facilitate adjusting the system across multiple dimensions. Ultimately, the best policy for maximizing progress toward the SDG targets in the context of small‐scale fisheries will likely be based on combining the range of strategies within the co‐management portfolio, using these in conjunction with other governance tools, and accepting that co‐management occurs within a much larger framework of political and socioeconomic conditions that also need to be considered and addressed. The solution is, therefore, ultimately a series of layers in governance and policy that are reactive to the social‐ecological system as it changes in these multiple dimensions.

## Supporting information

Supplementary materials
