# Supplementary material for: Linking small‐scale fisheries co‐management to U.N. Sustainable Development Goals
Source: Conserv Biol. 2022 Oct 19;36(6):e13977. doi: 10.1111/cobi.13977 (PMC10091792; doi:10.1111/cobi.13977)
Supplement: Supplementary file 1 — Supplementary materials [file COBI-36-0-s001.pdf]

Supplementary materials for:

Smallhorn-West, P., Cohen, P. J., Phillips, M., Jupiter, S. D., Govan, H., & Pressey, R. L. (2022) Linking small-scale fisheries co-management to U.N. Sustainable Development Goals. *Conservation Biology*.

Table S1. Evidence gap map (McKinnon et al. 2015) showing the studies included in this review which provide evidence for positive impacts from various small-scale fisheries co-management strategies for each of 11 U.N. Sustainable Development Goal (SDG) targets.

	Management strategy				
**SDG target**	**Access restrictions**	**Permanent closures**	**Periodic closures**	**Species restrictions**	**Gear restrictions**
**SDG 14.4** Resource status	Smallhorn-West et al. 2022	Albert et al. 2016; Bartlett et al. 2009; Bonaldo and Hay 2014; Bonaldo et al. 2017; Clements et al. 2012; Dumas et al. 2012; Januchowski-hartley et al. 2013; Jupiter et al. 2010; 2013; Jupiter and Egli 2011; Leopold et al. 2009; Rasher et al. 2013; Smallhorn-West et al. 2020; 2022; Tran et al. 2016	Bartlett et al. 2009; Cinner 2005; Cinner and McClanahan 2006; Cinner et al. 2019; Cohen and Alexander 2013; Cohen and Foale 2013; Dumas et al. 2012; Feary et al. 2011; Goetze et al. 2015; 2016; 2017; 2018; Januchowski-hartley et al. 2014 Jupiter et al. 2010; 2012; Jupiter and Egli 2011; McClanahan et al. 2006; Smallhorn-West et al. 2022	Almany et al. 2013; Foale 1998; Hamilton et al. 2011; Leopold et al. 2013; Nash et al. 1995	Cinner 2005; Lindfield et al. 2014; McClanahan et al. 2006; 2008 ; Naughton 1985; Veitayaki et al. 1995
**SDG 1.2** Livelihoods	0	Pascal 2009; Pascal and Seidle 2013; Weeks and Jupiter 2013; Beukering et al. 2014	Pascal and Seidle 2013	Leopold et al. 2013	0
**SDG 1.4/14B** Access rights	Dorset 2010; Foale et al. 2011; Johanness 2002; Macintyre and Foale 2007; Sloan and Chand 2016; Smallhorn-West et al. 2020	Weeks and Jupiter 2013; Smallhorn-West et al. 2020	Foale et al. 2011; Cohen and Steenbergen 2015	0	0

**SDG 5.5** Inclusivity	0	Egli et al. 2010	0	0	0
**SDG 11.4** Custom	Dorset 2010; Foale et al. 2011; Sloan and Chand 2016	0	Foale et al. 2011	Cappell and Lester 1953; Foale et al. 2011; Johannes 1978; Ravuvu 1983; Veitayaki 1995	0
**SDG 14.2** Ecosystem	0	Albert et al. 2016; Bonaldo and Hay 2014; Bonaldo et al. 2017; Clements and Hay 2017; Dell et al. 2015; 2016; Elgil et al. 2010; Jupiter et al. 2013; Rasher et al. 2013; Smallhorn-West et al. 2020	Jupiter et al. 2017	0	McClanahan et al. 2008; Naughton 1985; Veitayaki et al. 1995
**SDG 2.3** Short-term yield	0	0	Carvalho et al. 2019; Cinner 2005; Cinner et al. 2019; Cohen et al. 2013; Cohen and Foale 2013; Feary et al. 2011; Goetze et al. 2018; Januchowski-hartley et al. 2014; Smallhorn-West et al. 2022	Leopold et al. 2013; Nash et al. 1995	0
**SDG 2.4** Long-term yield	0	Pascal 2009; Pascal and Seidl 2013; Clements et al. 2012; Beukering et al.2014	Carvalho et al. 2019; Cinner et al. 2019; Smallhorn-West et al. 2022	Leopold et al. 2013	McClanahan et al. 2008
**SDG 2.1** Consumption	0	Aswani and Furusawa, 2007	0	0	0
**SDG 2.2** Nutrition	0	Aswani and Furusawa, 2007	0	0	0

References contained in figures, tables and supplementary materials:

Albert, S., Tawake, A., Vave, R., Fisher, P., & Grinham, A. (2016). Indicators of herbivorous fish biomass in community-based marine management areas in Fiji. *Pacific Conservation Biology*, *22*(1), 20-28.

Almany, G. R., Hamilton, R. J., Bode, M., Matawai, M., Potuku, T., Saenz-Agudelo, P., Planes, S., Berumen, M. L., Rhodes, K. L., Thorrold, S. R., Russ, G. R., & Jones, G. P. (2013). Dispersal of grouper larvae drives local resource sharing in a coral reef fishery. *Current Biology*, *23*(7), 626–630.

Aswani, S., & Furusawa, T. (2007). Do marine protected areas affect human nutrition and health? A comparison between villages in Roviana, Solomon Islands. *Coastal Management*, *35*(5), 545-565.Bartlett, C. Y., Pakoa, K., & Manua, C. (2009). Marine reserve phenomenon in the Pacific islands. *Marine Policy*, *33*(4), 673–678.

Beukering P, Scherl LM, Smith S, Hale L, Purvis N (2014) Natures investment banks. *Monogr Soc Res Child Dev* 79:141

Bjordal, Å. (2009). Regulation of fishing gears and methods. *A fishery manager's guidebook*, 165-195.

Bonaldo, R. M., & Hay, M. E. (2014). Seaweed-coral interactions: Variance in seaweed allelopathy, coral susceptibility, and potential effects on coral resilience. *PLoS ONE*, *9*(1), 30–34.

Bonaldo, R. M., Pires, M. M., Roberto, P., Hoey, S., & Hay, M. E. (2017). *Small Marine Protected Areas in Fiji Provide Refuge for Reef Fish Assemblages , Feeding Groups , and Corals*. 1–26.

Cappell, A., & Lester, R. H. (1953). The nature of Fijian totemism. *Fiji Society of Science and Industry*, *2*(1–5), 59–67.

Carvalho, P. G., Goetze, J., Jupiter, S. D., Claudet, J., Hartley, F. A. J., & Weeks, R. (2019). *Optimized fishing through periodically harvested closures*. *October 2018*, 1927–1936.

Cinner, J. (2005). Socioeconomic factors influencing customary marine tenure in the Indo-Pacific. *Ecology and Society*, *10*(1).

Cinner, J., & McClanahan, T. R. (2006). Socioeconomic factors that lead to overfishing in small-scale coral reef fisheries of Papua New Guinea. *Environmental Conservation*, 73–80.

Cinner, J. E., Graham, N. A. J., Huchery, C., & Macneil, M. A. (2012). *Global Effects of Local Human Population Density and Distance to Markets on the Condition of Coral Reef Fisheries*. *27*(3), 453–458.

Cinner, J. E., Maire, E., Huchery, C., MacNeil, M. A., Graham, N. A. J., Mora, C., McClanahan, T. R., Barnes, M. L., Kittinger, J. N., & Hicks, C. C. (2018). Gravity of human impacts mediates coral reef conservation gains. *Proceedings of the National Academy of Sciences*, *115*(27), E6116–E6125.

Cinner, J. E., Lau, J. D., Bauman, A. G., Feary, D. A., Januchowski-Hartley, F. A., Rojas, C. A., ... & Graham, N. A. J. (2019). Sixteen years of social and ecological dynamics reveal challenges and opportunities for adaptive management in

sustaining the commons. *Proceedings of the National Academy of Sciences*, *116*(52), 26474-26483.

Clements, C., Bonito, V., Grober-Dunsmore, R., & Sobey, M. (2012). Effects of small, Fijian community-based marine protected areas on exploited reef fishes. *Marine Ecology Progress Series*, *449*, 233-243.

Clements, C. S., & Hay, M. E. (2017). Size matters: Predator outbreaks threaten foundation species in small Marine Protected Areas. *PLoS One*, *12*(2), e0171569.

Cochrane, K. L., & Garcia, S. M. (2009). *A fishery manager's guidebook*. John Wiley & Sons.

Cohen, P. J., & Alexander, T. J. (2013). Catch rates, composition and fish size from reefs managed with periodically-harvested closures. *PLoS One*, *8*(9), e73383.

Cohen, P. J., & Foale, S. J. (2013). Sustaining small-scale fisheries with periodically harvested marine reserves. *Marine Policy*, *37*, 278–287.

Cohen, P. J., Cinner, J. E., & Foale, S. (2013). Fishing dynamics associated with periodically harvested marine closures. *Global Environmental Change*, *23*(6), 1702–1713.

Cohen, P. J., & Steenbergen, D. J. (2015). Social dimensions of local fisheries co-management in the Coral Triangle. *Environmental Conservation*, *42*(3), 278-288.

Dell, C., Montoya, J. P., & Hay, M. E. (2015). Effect of marine protected areas (MPAs) on consumer diet: MPA fish feed higher in the food chain. *Marine ecology progress series*, *540*, 227-234.

Dell, C. L., Longo, G. O., & Hay, M. E. (2016). Positive feedbacks enhance macroalgal resilience on degraded coral reefs. *PloS one*, *11*(5), e0155049.

Dorsett, S. (2010). The Act that almost was: the Fijian Qoliqoli Bill 2006. In *Comparative Perspectives on Communal Lands and Individual Ownership* (pp. 306–322). Routledge-Cavendish.

Dumas, P., Léopold, M., Kaltavara, J., William, A., Kaku, R., & Ham, J. (2012). Efficiency of tabu areas in Vanuatu. *Vanuatu Fisheries Department, Port Vila*.

Edgar, G. J., Stuart-smith, R. D., Willis, T. J., Kininmonth, S., Baker, S. C., Banks, S., Barrett, N. S., Becerro, M. A., Bernard, A. T. F., Berkhout, J., Buxton, C. D., Campbell, S. J., Cooper, A. T., Davey, M., & Edgar, S. C. (2014). Global conservation outcomes depend on marine protected areas with five key features. *Nature*, *506*, 216–220.

Egli, D. P., Tui, T., Jupiter, S. D., & Caginitoba, A. (2010). Perception surveys of coastal resource use and changes following establishment of a marine protected area network in Kubulau, Fiji. *Wildlife Conservation Society-Fiji Technical Report*, (07/10).

Feary, D. A., Cinner, J. E., Graham, N. A., & Januchowski-Hartley, F. A. (2011). Effects of customary marine closures on fish behavior, spear-fishing success, and underwater visual surveys. *Conservation Biology*, *25*(2), 341-349.

Foale, S. (1998). Assessment and management of the trochus fishery at West Nggela, Solomon Islands: An interdisciplinary approach. *Ocean and Coastal Management*, *40*(2–3), 187–205.

Foale, S., Cohen, P., Januchowski-Hartley, S., Wenger, A., & Macintyre, M. (2011). Tenure and taboos: Origins and implications for fisheries in the Pacific. *Fish and Fisheries*, *12*(4), 357–369.

Gelcich, S., Fernandes, M., Godoy, N., Canepa, A., Prado, L., & Castilla, J. C. (2012). Territorial User Rights for Fisheries as Ancillary Instruments for Marine Coastal Conservation in Chile. *Conservation Biology*, *26*(6), 1005–1015.

Gelcich, S., Cinner, J., Donlan, C. J., Tapia-Lewin, S., Godoy, N., & Castilla, J. C. (2017). Fishers' perceptions on the Chilean coastal TURF system after two decades: problems, benefits, and emerging needs. *Bulletin of Marine Science*, *93*(1), 53-67.Gelcich, S., Fernandes, M., Godoy, N., Canepa, A., Prado, L., & Castilla, J. C. (2012). Territorial User Rights for Fisheries as Ancillary Instruments for Marine Coastal Conservation in Chile. *Conservation Biology*, *26*(6), 1005–1015.

Gjertsen, H. (2005). Can habitat protection lead to improvements in human well-being? Evidence from marine protected areas in the Philippines. *World Development*, *33*(2 SPEC. ISS.), 199–217.

Goetze, J. S., Jupiter, S. D., Langlois, T. J., Wilson, S. K., Harvey, E. S., Bond, T., & Naisilisili, W. (2015). Diver operated video most accurately detects the impacts of fishing within periodically harvested closures. *Journal of Experimental Marine Biology and Ecology*, *462*, 74-82.

Goetze, J., Langlois, T., Claudet, J., Januchowski-hartley, F., & Jupiter, S. D. (2016). Periodically harvested closures require full protection of vulnerable species and longer closure periods. *BIOC*, *203*, 67–74.

Goetze, J. S., Januchowski-Hartley, F. A., Claudet, J., Langlois, T. J., Wilson, S. K., & Jupiter, S. D. (2017). Fish wariness is a more sensitive indicator to changes in fishing pressure than abundance, length or biomass. *Ecological Applications*, *27*(4), 1178–1189.

Goetze, J. S., Claudet, J., Januchowski-Hartley, F., Langlois, T. J., Wilson, S. K., White, C., Weeks, R., & Jupiter, S. D. (2018). Demonstrating multiple benefits from periodically harvested fisheries closures. *Journal of Applied Ecology*, *55*(3), 1102–1113.

Govan, H., Aalbersberg, W., Tawake, A., & Parks, J. (2008). *Locally managed marine areas: A guide to supporting community-based adaptive management*.

Hamilton, S. L., Caselle, J. E., Standish, J. D., Schroeder, D. M., Love, M. S., Rosales-Casian, J. A., & Sosa-Nishizaki, O. (2007). Size-selective harvesting alters life histories of a temperate sex-changing fish. *Ecological Applications*, *17*(8), 2268–2280.

Hamilton, R. J., Potuku, T., & Montambault, J. R. (2011). Community-based conservation results in the recovery of reef fish spawning aggregations in the Coral Triangle. *Biological Conservation*, *144*(6), 1850–1858.

Harrison, H. B., Williamson, D. H., Evans, R. D., Almany, G. R., Thorrold, S. R., Russ, G. R., Feldheim, K. A., van Herwerden, L., Planes, S., & Srinivasan, M. (2012). Larval export from marine reserves and the recruitment benefit for fish and fisheries. *Current Biology*, *22*(11), 1023–1028.

Januchowski-Hartley, F. A., Graham, N. A. J., Cinner, J. E., & Russ, G. R. (2013). Spillover of fish naïveté from marine reserves. *Ecology Letters*, *16*(2), 191–197.

Januchowski-hartley, F. A., Cinner, J. E., & Graham, N. A. J. (2014). *Fishery benefits from behavioural modification of fishes in periodically harvested fisheries closures*. *790*(August 2013), 777–790.

Johannes, R. E. (1978). Traditional marine conservation methods in Oceania and their demise. *Annu. Rev. Ecol. Syst.*, *9:349–64*.

Johannes, R. E. (2002). The renaissance of community-based marine resource management in Oceania. *Annual Review of Ecology and Systematics*, *33*, 317–340.

Jupiter, S. D., Egli, D. P., Jenkins, A. P., Yakub, N., Hartley, F., Cakacaka, A., ... & Prasad, S. (2010). Effectiveness of marine protected area networks in traditional fishing grounds of Vanua Levu, Fiji, for sustainable management of inshore fisheries. *Wildlife Conservation Society-Fiji and Wetlands International-Oceania Technical Report*, *3*(10).

Jupiter, S. D., & Egli, D. P. (2011). *Ecosystem-Based Management in Fiji : Successes and Challenges after Five Years of Implementation*. *2011*. https://doi.org/10.1155/2011/940765

Jupiter, S. D., Weeks, R., Jenkins, A. P., Egli, D. P., & Cakacaka, A. (2012). Effects of a single intensive harvest event on fish populations inside a customary marine closure. *Coral Reefs* 321–334.

Jupiter, S., Saladrau, W., & Vave, R. (2013). Assessment of sea cucumber fisheries through targeted surveys of Lau Province, Fiji. *Wildlife Conservation Society/University of the South Pacific/Fiji Department of Fisheries/Khaled bin Sultan Living Oceans Foundation, Suva, Fiji*.

Jupiter, S. D., Epstein, G., Ban, N. C., Mangubhai, S., Fox, M., Cox, M., Jupiter, S. D., Epstein, G., Ban, N. C., & Mangubhai, S. (2017). A Social – Ecological Systems Approach to Assessing Conservation and Fisheries Outcomes in Fijian Locally Managed Marine Areas. *Society & Natural Resources*, *30*(9), 1096–1111.

Kawarazuka, N. (2010). The contribution of fish intake, aquaculture, and small-scale fisheries to improving food and nutrition security: A literature review. The WoldFish Working Paper No.2106. The WoldFish Centre, Penang , Malaysia. 51 p. *The WorldFish Center Working Paper*, 44.

Léopold, M., Cakacaka, A., Meo, S., Sikolia, J., & Lecchini, D. (2009). Evaluation of the effectiveness of three underwater reef fish monitoring methods in Fiji. *Biodiversity and Conservation*, *18*(13), 3367-3382.

Léopold, M., Beckensteiner, J., Kaltavara, J., Raubani, J., & Caillon, S. (2013). Community-based management of near-shore fisheries in vanuatu: What works? *Marine Policy*, *42*, 167–176.

Lindfield, S. J., McIlwain, J. L., & Harvey, E. S. (2014). Depth refuge and the impacts of SCUBA spearfishing on coral reef fishes. *PLoS ONE*, *9*(3), 1–12.

Macintyre, M., & Foale, S. (2007). Land and marine tenure, ownership, and new forms of entitlement on Lihir: changing notions of property in the context of a goldmining project. *Human Organization*, *66*(1), 49–59.

McClanahan, T. R., Marnane, M. J., Cinner, J. E., & Kiene, W. E. (2006). A Comparison of Marine Protected Areas and Alternative Approaches to Coral-Reef Management. *Current Biology*, *16*(14), 1408–1413.

McClanahan, T., Sebastián, C., & Cinner, J. (2008). Managing fishing gear to encourage ecosystem-based management of coral reefs fisheries. *Proc 11th Int Coral Reef …*, *July*, 7–11. http://www.nova.edu/ncri/11icrs/proceedings/files/m22-06.pdf%5Cnhttp://41.215.122.106/dspace/handle/0/2893

McClanahan, T. R. (2010). Effects of fisheries closures and gear restrictions on fishing income in a Kenyan coral reef. *Conservation Biology*, *24*(6), 1519–1528.

McClanahan, T. R., & Hicks, C. C. (2011). Changes in life history and ecological characteristics of coral reef fish catch composition with increasing fishery management. *Fisheries Management and Ecology*, *18*(1), 50–60.

McClanahan, T. R. (2021). Marine reserve more sustainable than gear restriction in maintaining long-term coral reef fisheries yields. *Marine Policy*, *128*, 104478.

Nash, W., Adams, T., Tuara, P., Terekia, O., Munro, D., Amos, M., ... & Whitford, J. (1995). *The Aitutaki trochus fishery: a case study*. South Pacific Commission.

Naughton, J. (1985). Blast fishing in the Pacific. *SPC Fisheries Newsletter*, *33*, 16–20.

Pascal, N. (2009). *Cost-benefi t analysis of community-based marine protected areas: Five case studies in Vanuatu*. *1*, 41–48.

Pascal, N., & Seidle, A. (2013). *Economic benefits of marine protected areas: case studies in Vanuatu and Fiji, South Pacific. In: Research report, AFD/IUCN. French Framework Agreement by CRIOBE (EPHE/ CNRS), Mooréa, French Polynesia*.

Rasher, D. B., Hoey, A. S., & Hay, M. E. (2013). Consumer diversity interacts with prey defenses to drive ecosystem function. *Ecology*, *94*(6), 1347-1358.

Ravuvu, A. (1983). *Vaka i Taukei: The Fijian way of life*. Institute of the Pacific Studies.

Russ, G. R. (2002). Yet another review of marine reserves as reef fishery management tools. *Coral Reef Fishes: Dynamics and Diversity in a Complex Ecosystem*, *24*, 421.

Russ, G. R., & Alcala, A. C. (1996). Marine reserves: rates and patterns of recovery and decline of large predatory fish. *Ecological Applications*, *6*(3), 947–961.

Russ, G. R., & Alcala, A. C. (2004). Marine reserves: Long-term protection is required for full recovery of predatory fish populations. *Oecologia*, *138*(4), 622–627. https://doi.org/10.1007/s00442-003-1456-4

Sloan, J., & Chand, K. (2016). An analysis of property rights in the Fijian qoliqoli. *Marine Policy*, *72*, 76–81.

Smallhorn-West, P. F., Weeks, R., Gurney, G., & Pressey, R. L. (2020a). Ecological and socioeconomic impacts of marine protected areas in the South Pacific: assessing the evidence base. *Biodiversity and Conservation*, *29*(2).

Smallhorn-West, P. F., Stone, K., Ceccarelli, D., MaliMali, S., Halafihi, T., Bridge, T., Pressey, R., & Jones, G. (2020b). Community management yields positive impacts for coastal fisheries reosurces and biodiversity conservation. *Conservation Letters*, *e12755*. 10.1111/conl.12755

Smallhorn-West, P., Cohen, P., Morais, R., Januchowski-Hartley, F., Ceccarelli, D., Malimali, S., ... & Cinner, J. (2022). Hidden benefits and risks of partial protection for coral reef fisheries. *Ecology and Society*, *27*(1).

Tran, D. S. C., Langel, K. A., Thomas, M. J., & Blumstein, D. T. (2016). Spearfishing-induced behavioral changes of an unharvested species inside and outside a marine protected area. *Current Zoology*, *62*(1), 39–44.

Veitayaki, J., Ram-Bidesi, V., Matthews, E., Gibson, L., & Vuki, V. (1995). *South Pocific. Regionol Environment Progromme Ovenriew of Destructive Fishing Practices in the Pacific Islands Region*. *93*.

Vunisea, A. (2008). The "culture of silence" and fisheries management. *SPC Women in Fisheries Information Bulletin*, *18*(March), 42–43.

Weeks, R., & Jupiter, S. D. (2013). Adaptive comanagement of a marine protected area network in Fiji. *Conservation Biology*, *27*(6), 1234-1244.

Wilson, D. C., Ahmed, M., Siar, S. v., & Kanagaratnam, U. (2006). Cross-scale linkages and adaptive management: Fisheries co-management in Asia. *Marine Policy*, *30*(5), 523–533.
